# Electric Field-Assisted In Situ Precise Deposition of Electrospun γ-Fe_2_O_3_/Polyurethane Nanofibers for Magnetic Hyperthermia

**DOI:** 10.1186/s11671-018-2707-y

**Published:** 2018-09-10

**Authors:** Chao Song, Xiao-Xiong Wang, Jun Zhang, Guang-Di Nie, Wei-Ling Luo, Jie Fu, Seeram Ramakrishna, Yun-Ze Long

**Affiliations:** 1 Collaborative Innovation Center for Nanomaterials & Devices, College of Physics, Qingdao University, Qingdao, 266071 China; 2 Industrial Research Institute of Nonwovens & Technical Textiles, College of Textiles & Clothing, Qingdao University, Qingdao, 266071 China; 3 Center for Nanofibers & Nanotechnology, Faculty of Engineering, National University of Singapore, Singapore, Singapore

**Keywords:** Electrospinning, In situ precise deposition, Auxiliary electrode, Magnetic hyperthermia

## Abstract

A facial electrospinning method of in situ precise fabricating magnetic fibrous membrane composed of polyurethane (PU) nanofibers decorated with superparamagnetic γ-Fe_2_O_3_ nanoparticles with simultaneous heat generation in response to alternating magnetic field (AMF) is reported. In this method, a conical aluminum auxiliary electrode is used to regulate the electrostatic field and affect the process of electrospinning for the in situ rapid and precise deposition of electrospun γ-Fe_2_O_3_/PU fibers. The auxiliary conical electrode can extend the jet stabilization zone of the precursor solution four times longer than that of without auxiliary electrode, which can achieve the precise control of the fiber deposition area. Moreover, the electrospun composite fibrous membranes show a rapid temperature increase from room temperature to 43 °C in 70 s under the AMF, which exhibits faster heating rate and higher heating temperature compared to the samples fabricated without the assist of the auxiliary electrode. The present results demonstrate that the in situ precise electrospinning with the help of an auxiliary conical electrode has the potential as a manipulative method for preparing magnetic composite fibers as well as magnetic hyperthermia of cancer therapy.

## Background

Hyperthermia is another effective treatment after the traditional treatment of tumor, which can be used in multimodality strategies, as it can synergistically enhance the antitumor effects of chemotherapy, radiotherapy, and immunotherapy [1–3]. The mechanism of using hyperthermia as a treatment measure for cancer is the sensitivity of cancer cells to the range from 41 to 45 °C, in contrast to healthy cells [4, 5]. The application of magnetic nanoparticles for cancer diagnosis and therapy is still limited by poor orientation although many targeting strategies have been performed, such as magnetic targeting and molecular targeting [6, 7]. If the magnetic particles are injected directly into the body, they are quickly cleared by the reticuloendothelial system and enriched in certain organs such as the kidney, liver, and spleen rather than the tumor site [8]. Furthermore, the magnetic nanoparticles are locally injected in the tumor tissue and they prefer to leak out from the tumor site due to their small size [9]. All of these cases lower the hyperthermia efficacy of magnetic nanoparticles. Compared to the magnetic nanoparticles hyperthermia, the precise and local delivery of iron oxide nanoparticles (INOPs) to cancer cells is the main advantage of magnetic composite fibers, which is an ideal hyperthermia treatment method for easily accessible tumors.

In many reports, composite nanofibrous membranes were prepared from a polymer solution containing a dispersion of previously synthesized magnetic nanoparticles before it, which were employed in hyperthermia treatment [10–13]. However, all of them have some obvious drawbacks. For example, it is difficult for the previously prepared nanofibers to develop a homogeneous and continuous coating on the surface of the tumor tissue, which is easy to break off and directly adverse to a tight seal, and then leads to the inadequacy in complex tumor tissue. As a result, the in situ precise electrospinning onto the tumor tissue could be a good strategy not only for preventing the peeling of composite fibrous membranes during the magnetic hyperthermia process, but also for increasing the chance for homogeneous heating onto the tumor tissue.

The in situ electrospinning needs to precisely control the deposition range on a specified tumor tissue, which could avoid causing serious tissue adhesion especially in the abdominal cavity [14]. It has been reported by many researchers that the in situ precise electrospinning can be applied in the field of medicine [15]. Recent studies demonstrated the use of airflow-directed in situ electrospinning device to enhance the precise deposition of ultrathin polymeric fibers onto wound sites [16]. However, airflow-directed in situ electrospinning device need to add an extra air pump, an additional home-made handle with a spinneret head, and other air flow-assisted device compared to the traditional electrospinning apparatus, and need to coordinate the air flow and the relationship between the speed and voltage, which makes the operation of the apparatus complicated. Magnetic field-assisted electrospinning is also an effective way to prepare ordered electrospun fibers and control the deposition range of electrospun fibers [17]. The macromolecular polymers incorporating enough magnetic particles can induce sufficient magnetic susceptibility to the polymeric solution. Yang et al. [18] reported an approach for fabrication of well-aligned arrays and multilayer grids by a method called magnetic electrospinning (MES). In the MES, a small amount of magnetic nanoparticles is added to magnetize the polymer solution. A magnetic field generated by two parallel positioned permanent magnets is applied during electrospinning, and the magnetic field stretches the fibers across the gap to form a parallel array. However, the magnetic field-assisted electrospinning can only be used for the preparation of magnetic fibers, which has no effect on the spinning process for non-magnetic fibers. In contrast, the use of an auxiliary electrode in the in situ precise electrospinning to achieve the magnetic composite fibrous membranes is a more simple, effective, and universal method.

Here, we developed an in situ precise deposition of composite fibrous membranes with embedded γ-Fe_2_O_3_ NPs by a portable electrostatic spinner which adds a conical aluminum auxiliary electrode at the location of the spinning head to regulate the deposition direction and extent of magnetic composite fibers. The present study aims to explore the influence of the auxiliary electrode on the magnetic composite fibrous membranes whose morphology and performance may be changed. Compared with the conventional approach, this technique could very rapidly, precisely deposit nanostructured fibers on complex, irregular tumor tissue to form continuous, compact, flexible membranes with excellent integrity, which acts as a powerful source to locally heat the tumor region to the desired temperature without overheating the surrounding healthy tissues and to prevent possible tumor growth or enhance the antitumor effects of chemotherapy, radiotherapy, and immunotherapy. In vitro studies showed that the electrospun γ-Fe_2_O_3_/PU magnetic fiber membranes have excellent magnetic-mediated hyperthermia therapeutic efficacy. Furthermore, the heat stability of γ-Fe_2_O_3_/PU composite fibrous membranes has also been demonstrated by the repeated magnetic field excitation.

## Methods/Experimental

### Materials

γ-Fe_2_O_3_ nanoparticles (γ-Fe_2_O_3_ NPs, 10 nm, phere, 99.5%, Shanghai Macklin Biochemical Co., Ltd. China), high molecular weight polyether grade polyurethane pellets (PU, WHT-8170, Yantai Wanhua Polyurethanes Co., Ltd., China), and *N*,*N*-dimethylformamide (DMF ≥ 99.5%, Pharm Chemical Reagent Co, Ltd., China) were used as received without further purity.

### Preparation of Electrospun γ-Fe_2_O_3_/PU Magnetic Nanofibers

In order to disperse γ-Fe_2_O_3_ NPs in DMF, 0.54-g nanopowder was added into 2.5-g DMF, after which the mixture was exposed to ultrasonic for 4 h in a cone bottle. Neat polyurethane solution was prepared by dissolving 1.8-g PU pellets in 7.5-g DMF solvent and stirring at 40 °C. Then, the PU solution was poured into the γ-Fe_2_O_3_ NPs dispersion and vigorously stirred for 30 min by a homemade mechanical stirrer. Finally, the solution mixture was further dispersed by sonication for another 24 h at 50 °C before electrospinning.

**Fig. 1:**
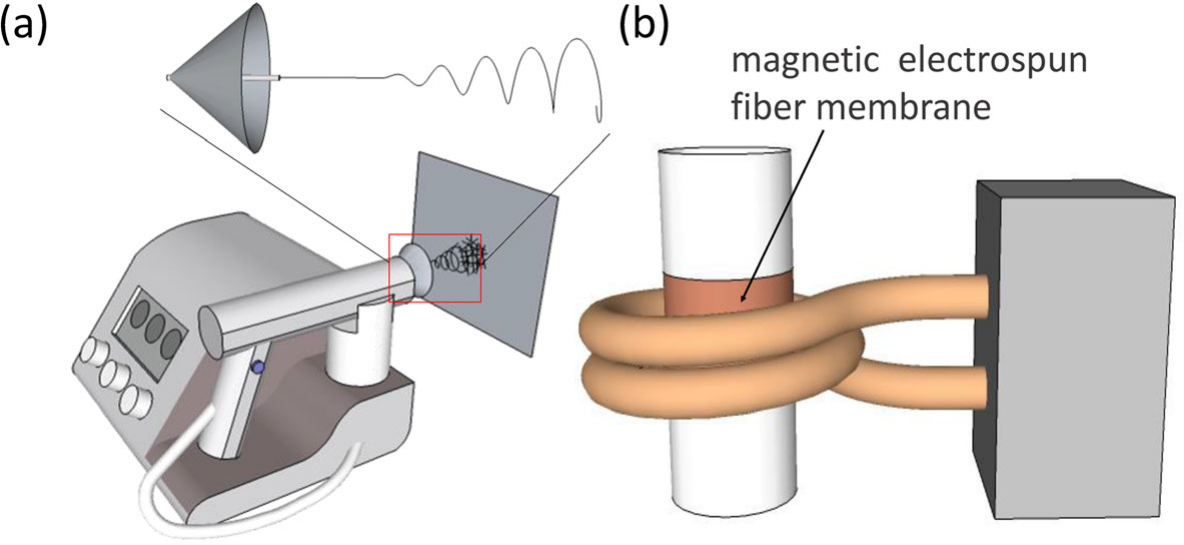
**a** Schematic diagram of portable electrostatic spinning device and auxiliary electrode. **b** Schematic representation of the field generator, coils, and data acquisition system for recording temperature

In the electrospinning process, we adopted a portable electrostatic spinner to achieve the manufacture of magnetic fibers. As shown in Fig. 1a, the portable electrostatic spinner has a gun-shaped hand-held spinning device. As shown in the enlarged image of the spinning head, a conical auxiliary electrode with a diameter of 4 cm on the bottom was fixed at the spinneret, which can control and regulate electrostatic field for the rapid deposition of fibers to the ideal thickness. This electrospinning device used a 5-mL plastic syringe (Becton Dichinson), to which a needle tip with an inner diameter of 0.7 mm was attached. The electrospinning precursor solution was loaded in the syringe and squeezed by a syringe pump of the device. Electrospinning was carried out at an applied voltage varying between 10~ 15 kV, with an apex-to-collector distance of 10 cm and a constant feed rate of 33 μL/min. The collector could be aluminum foil, the skin and even the surface of tumor tissue. Under the effect of electrostatic field force, the electrospinning precursor solution squeezed by a syringe pump was stretched, cleaved to nanofiber in the air, and eventually deposited on the surface of collector. After different spinning time of 5, 10, 15, and 20 min, different thicknesses of the magnetic fiber membranes were obtained and denoted as γ-Fe_2_O_3_/PU-A5, γ-Fe_2_O_3_/PU-A10, γ-Fe_2_O_3_/PU-A15, and γ-Fe_2_O_3_/PU-A20, respectively. Moreover, the γ-Fe_2_O_3_/PU composite fibrous membranes were also prepared in the same electrospinning time and conditions but without the auxiliary electrode during electrospinning and were denoted as γ-Fe_2_O_3_/PU-5, γ-Fe_2_O_3_/PU-10, γ-Fe_2_O_3_/PU-15, and γ-Fe_2_O_3_/PU-20, respectively. All procedures were carried out according to the guidelines of the National Institutes of Health for use of laboratory animals and with the approval of the Chancellor’s Animal Research Committee at the University.

### Characterization

The surface morphological structure and diameters of the γ-Fe_2_O_3_/PU composite nanofibrous membranes were determined by scanning electron microscopy (SEM, TM-1000, Hitachi). The size and distribution of magnetic particles were characterized using a transmission electron microscope (TEM, JEM-200EX). X-ray powder diffraction (XRD, RINT2000 wide angel goniometer) analysis was carried out by using a Rigaku X-ray diffractometer. The chemical composition and molecular structure of the nanofiber membranes were determined by using a Fourier transformer infrared (FTIR) spectrometer (Thermo Scientific Nicolet iN10). Thermogravimetric analysis (TGA) of the composition membranes was performed at a heating rate of 10 °C/min from 30 to 600 °C under the protection of nitrogen flow. Magnetic properties of γ-Fe_2_O_3_/PU were measured by the vibration sample magnetometer (VSM, Quantum Design Corporation) from − 15,000 to 15,000 Oe.

### Magnetic Heating Experiment

The excited AMF used to induce the heating treatment was produced by an alternating magnetic field generator (SP-04AC Shenzhen Shuangping Power Technology Co., Ltd.) whose rated power was 3 kW and water-cooled induction coil was made of copper, with a two-tuning coil and an inner diameter of 30 mm (Fig. 1b). The maximum magnetic field intensity of the AMF generator and the magnetic field frequency were 12.5 Oe and 153 kHz, respectively. The fibrous membranes in a cylindrical form were placed in the center of the copper coil [19, 20]. To measure the heating characteristics of fibrous membranes, the infrared temperature detector was fixed over the fiber membranes and the temperature change of the nanofiber films was recorded in real time.

## Result and Discussion

### Precise Deposition via the In Situ Electrospinning

**Fig. 2:**
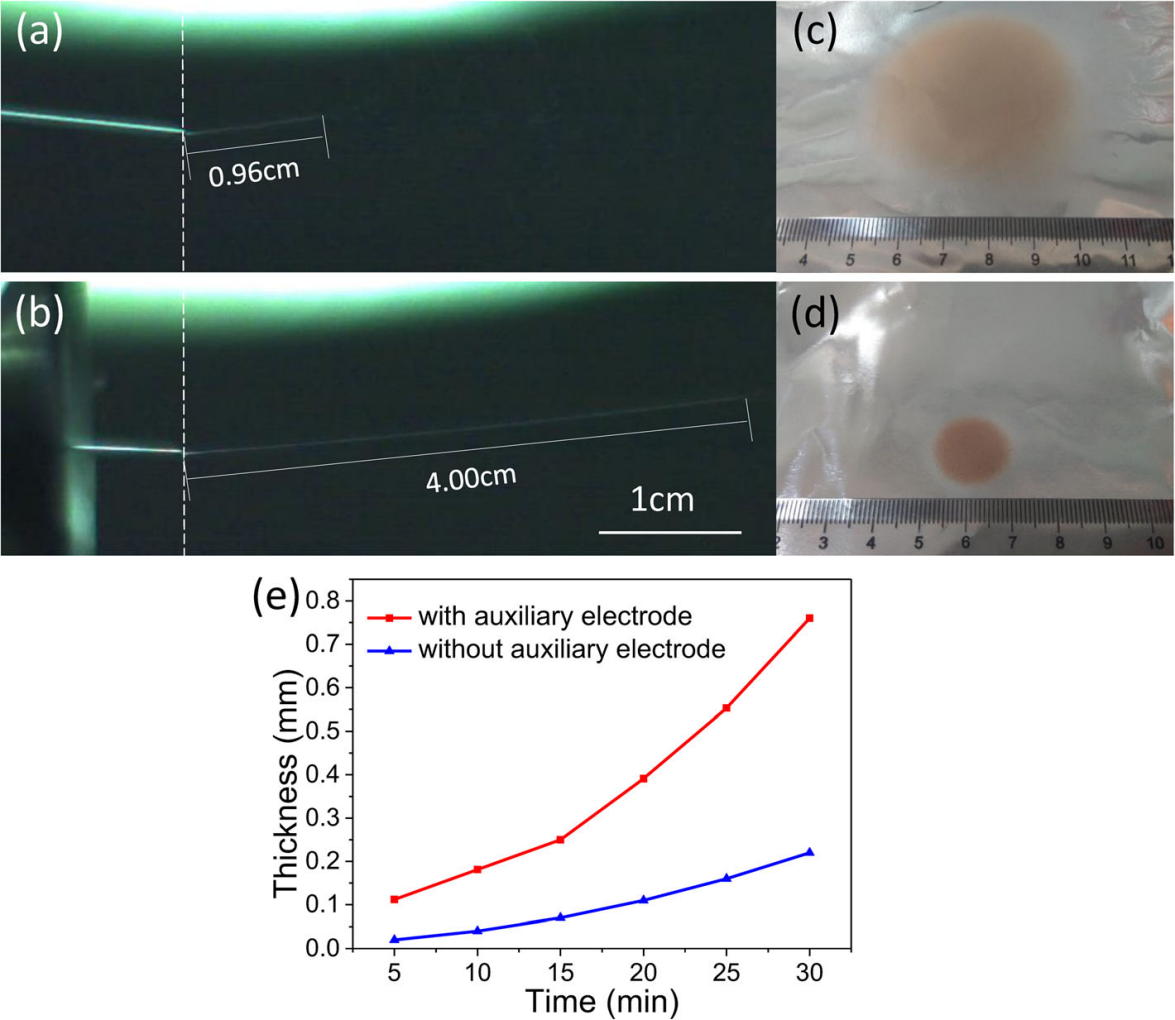
High-speed camera photos of electrospinning jet at steady zone **a** without and **b** with the auxiliary electrode. Optical photographs of in situ deposited electrospun fiber membrane **c** without and **d** with the auxiliary electrode. **e** Time-dependent deposition thickness curves for in situ preparation of electrospun fiber membranes

A comparison in the deposition range between electrospinning with and without an auxiliary electrode was performed. As shown in Fig. 2, under the same external conditions (temperature, voltage, distance, humidity, spinning speed, spinning precursor fluid, spinning needle diameter, etc.), the deposition range of the fibrous membrane prepared using the auxiliary electrode (diameter of 1.8 cm) was significantly smaller than that of the electrospun fiber without the use of the auxiliary electrode (diameter of 4.6 cm). In the traditional electrospinning process, the spinning precursor fluid splits, whips, and stretches, into micro-/nanoscale fiber in the air, and finally deposits on the collector to form a non-woven fabric membrane [21]. However, in the unstable region of the spinning jet, the conical spatial distribution of the jet increases the deposition range of the fiber and reduces the accuracy of the fiber deposition. When modified with an auxiliary electrode, the splitting and whipping of the spinning precursor jet are suppressed and the range of the jet stabilization region becomes large and fluctuates in a very narrow lane. As shown in Fig. 2a, b, without the help of the auxiliary electrode, the jet stabilization zone of the precursor solution was 0.96 cm. And with the aid of the auxiliary electrode, the jet stabilization zone of the precursor solution was extended by 4 cm, which was four times longer than that of without the auxiliary electrode. At the same spinning distance, the extension of the stabilization zone helps to reduce the spinning deposition range and achieve the in situ precise spinning. As shown in Fig. 2c, d, the deposition ranges of composite fibrous membranes prepared without and with the aid of an auxiliary electrode are circular regions whose diameters are 4.6 and 1.8 cm, respectively. The result demonstrates that the auxiliary electrode can effectively reduce the deposition range during the electrospinning process. Figure 2e shows the trend of the thickness of the electrospun fiber membrane over time. With the help of the auxiliary electrode, rapid electrospinning can be achieved, and after 30 min, the thickness of the deposited composite membrane is about four times thicker than that prepared by other electrospinning method. It is clear that under the mediation of the auxiliary electrode, the electrospinning jet has a more precise deposition range and a fibrous film having a certain thickness can be formed in a short time, which has great significance in executing the in situ precise spinning and realizing the rapid electrospinning in the following magnetic hyperthermia experiment.

### Morphological, Structural, and Magnetic Properties

**Fig. 3:**
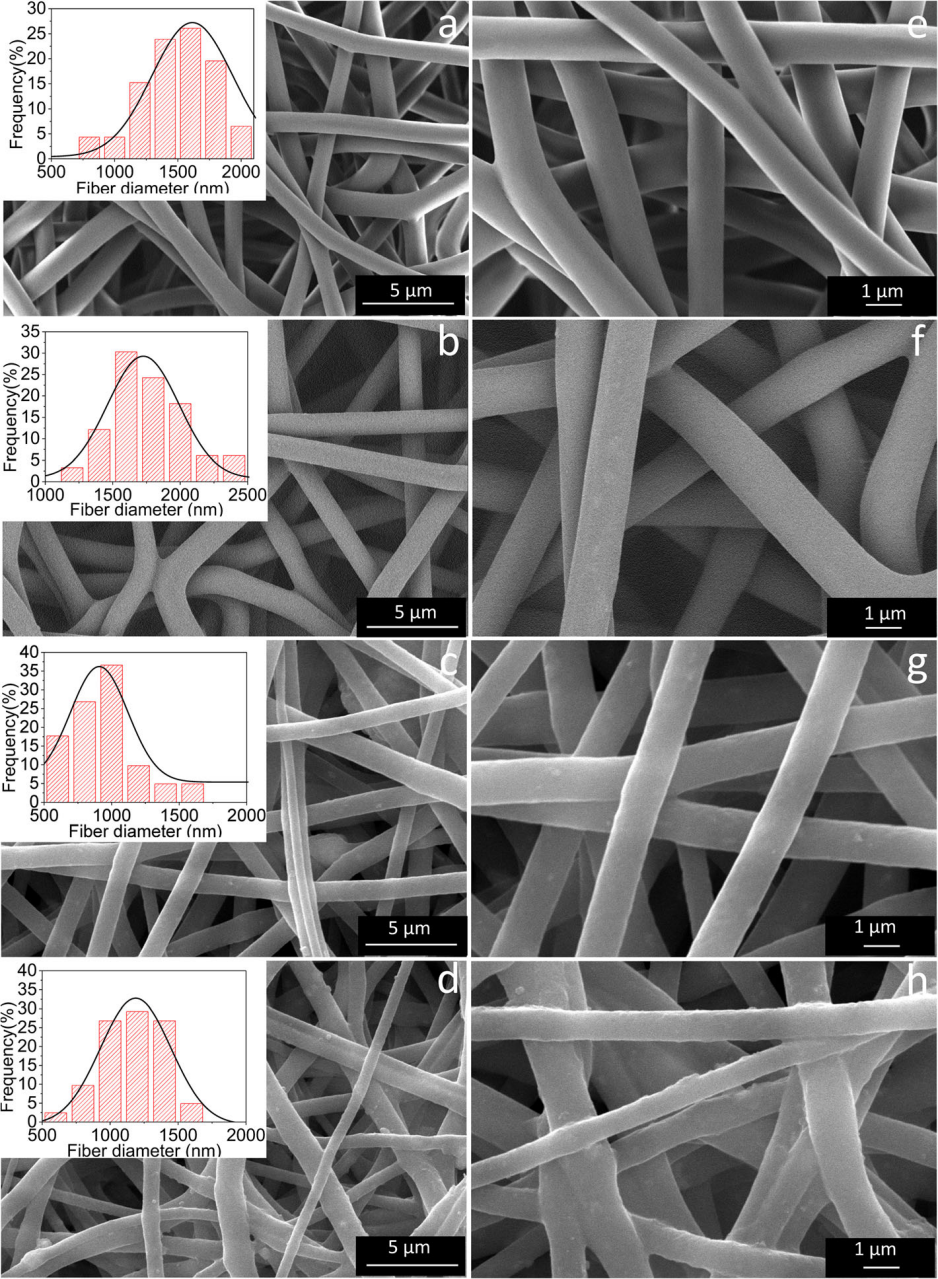
SEM images of pure PU fiber membranes prepared **a**, **e** without and **b**, **f** with the use of an auxiliary electrode. γ-Fe_2_O_3_/PU composite fiber membranes prepared **c**, **g** without and **d**, **h** with the use of an auxiliary electrode (the insets show the diameter distributions of the electrospun fiber membranes)

The SEM images of PU fibrous membranes and composite membranes prepared with/without an auxiliary electrode are shown in Fig. 3. As is apparent from Fig. 3a, e, b, and f, the PU fibrous membranes with sub-micro size, high porosity, and random ordered orientation prepared with and without an auxiliary electrode are both the relatively bead-free and smooth matrixes of interlocking fibers. According to the statistical analyses that are inserted in the upper left corners of the SEM images, the ranges of diameters of PU fibrous membranes which are prepared in two different ways are 700–1900 nm and 1100–2300 nm, respectively, and the mean fiber diameters of them are about 1390 and 1670 nm, respectively. Obviously, the fiber diameters of PU fibrous membranes prepared with an auxiliary electrode are a little thicker than those of the other, which could be attributed to the restriction of electrostatic field by an auxiliary electrode. The addition of the auxiliary electrode constrains the electric field and further limits the whipping and elongation of the spun fibers, so that the spun fibers are relatively thicker than those fabricated in the way where the auxiliary electrodes are not added. As showed in Fig. 3c, g, d, and h, the addition of γ-Fe_2_O_3_ NPs slightly changes the surface morphology and diameter of fibers, but it does not change the geometry and porous structure of the composite fibrous membranes in comparison to PU. After the incorporation of γ-Fe_2_O_3_ NPs, the fiber diameter was reduced to 850 nm and the surface of fibers exhibited an increased roughness, which might be due to the dispersion of γ-Fe_2_O_3_ NPs in/on the PU fibers because of their high surface-to-volume ratio [22]. However, the as-prepared composite fibrous membranes using an auxiliary electrode become less smooth (Fig. 3d). Therefore, in addition to the effects of magnetic particles, the addition of the auxiliary electrode during the electrospinning process inhibits the whipping of the fibers, and the solvent volatilization is incomplete, causing the fiber surface to become rougher. After the addition of γ-Fe_2_O_3_ nanoparticles, besides the change in the microscopic morphology of the nanofibers, the color of the composite nanofiber membranes also changed from white to light brown, and the color remained unchanged after several washings.

**Fig. 4:**
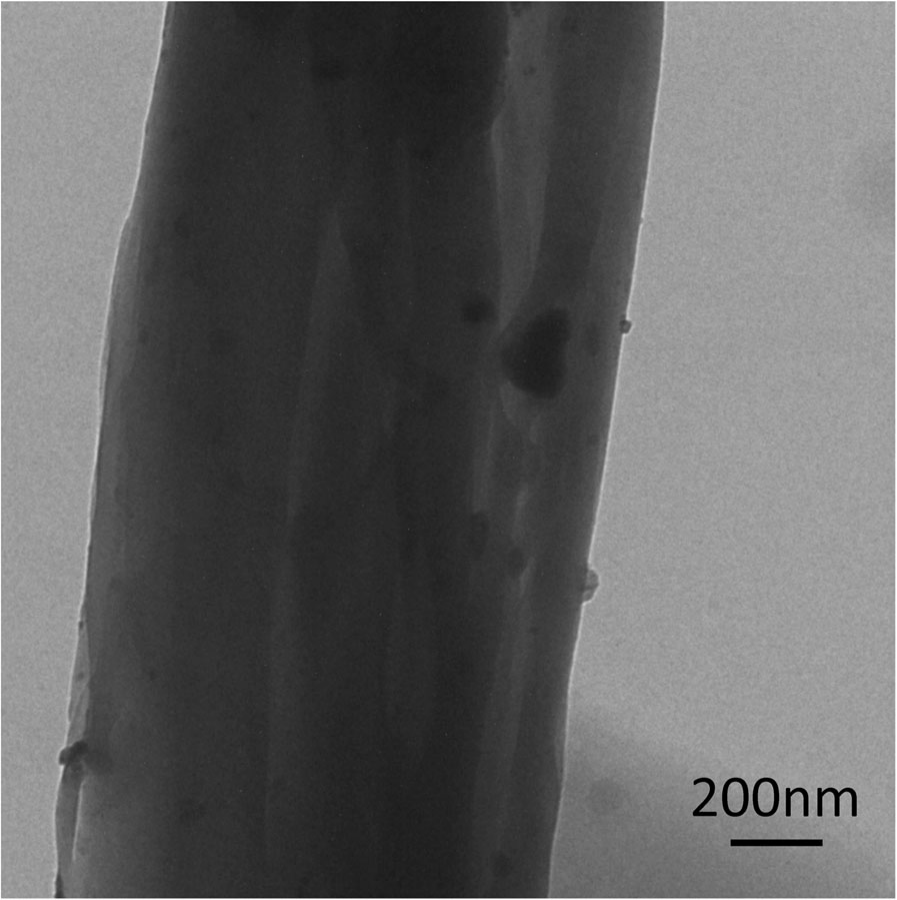
TEM images of γ-Fe_2_O_3_/PU composite fiber membranes prepared with the use of an auxiliary electrode

In order to further characterize the dispersion of γ-Fe_2_O_3_ NPs incorporated in the magnetic membranes, we have analyzed the TEM image of the composite fiber membrane in detail. As can be observed in Fig. 4, the γ-Fe_2_O_3_ NPs are well dispersed and the majority of them are incapsulated firmly inside the nanofibers, thus preventing their possible leakage and migration when used as the substrate materials for magnetic hyperthermia. The γ-Fe_2_O_3_ NPs show good dispersion and no agglomeration in the fiber, which means that the auxiliary electrode does not interfere the uniform distribution of magnetic particles.

**Fig. 5:**
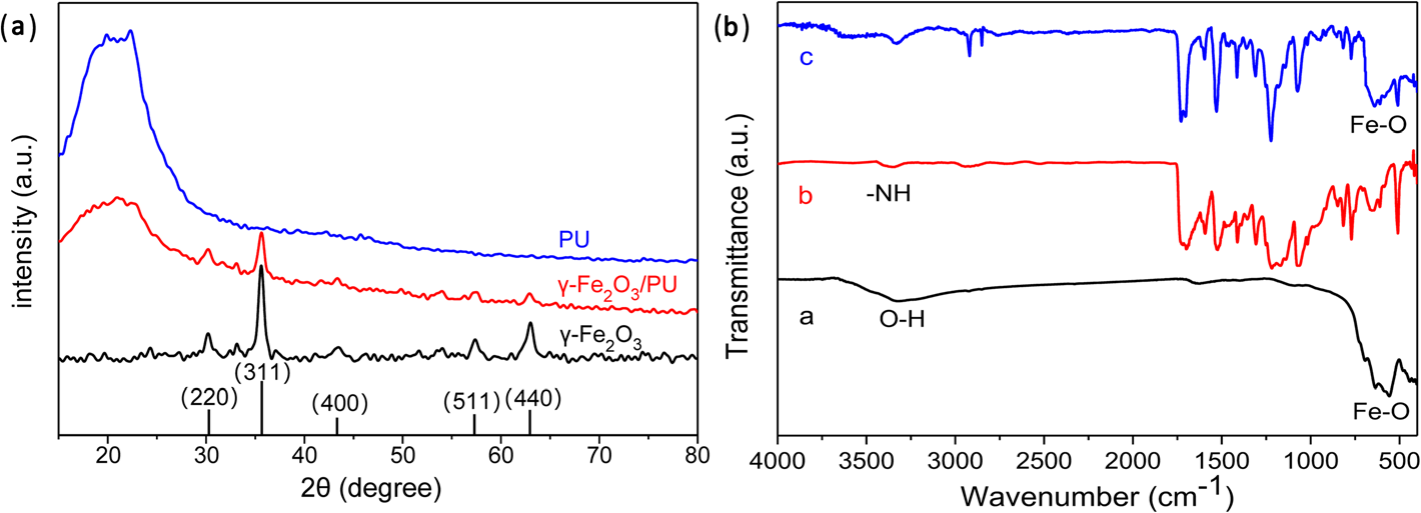
**a** XRD patterns of PU nanofibers, γ-Fe_2_O_3_/PU composite fiber membranes and γ-Fe_2_O_3_ NPs. **b** FTIR spectra of (*a*) γ-Fe_2_O_3_ NPs, (*b*) PU electrospun fiber membranes, and (*c*) magnetic composite fiber membranes

Figure 5A shows the XRD patterns of neat PU fiber membranes, γ-Fe_2_O_3_ magnetic nanoparticles, and electrospun γ-Fe_2_O_3_/PU composite fibrous membranes. It is found that the XRD spectra of the electrospun γ-Fe_2_O_3_/PU composite nanofibrous membranes and neat PU fiber membranes display one broad peak, pointing out a typical symbol for low crystalline materials. This result proves that the prepared PU fibrous membrane has low crystallinity. However, the positions and relative intensities of some new peaks of the composite membrane agree well with the standard diffraction card JCPDS 39-1346, which are corresponding to (220), (311), (400), (511), and (440) characteristic peaks of γ-Fe_2_O_3_ magnetic nanoparticles. Compared with the γ-Fe_2_O_3_ NPs, the significant decrease of the diffraction peak intensity of composite fibrous membranes can be attributed to the physical combination between γ-Fe_2_O_3_ NPs and PU fibrous membranes without chemical reaction.

To determine the molecular structure of the composite fibrous membranes, Fourier transform infrared (FTIR) spectra of the samples were analyzed in the spectral range of 400–4000 cm^−1^ (Fig. 5B). The main band assignments are listed in Table 1. The curve a in Fig. 5B presents a weak and broad absorption peak observed around 3347 cm^−1^, corresponding to the O-H stretching vibration of H_2_O due to the moisture absorption in γ-Fe_2_O_3_ NPs. In addition, a strong band at 557 cm^−1^ can be assigned to the vibrational frequency of the Fe-O bond. As shown by the curve b in Fig. 5B, the strong absorption band of electrospun PU membranes at 3328 cm^−1^ can be attributed to the N-H stretching; the band at 2919 cm^−1^ is assigned to the stretching vibration of the C-H bond in PU; the bands at 1704, 1729, 1529, 1073, and 771 cm^−1^ arise from the C-H asymmetrical flexing vibration, >C=H stretching vibrations, amide II band, C-O stretching, and CH_2_ rocking, respectively [23–25]. On the other hand, by comparison, the curve c in Fig. 5B shows the phenomenon that when γ-Fe_2_O_3_ NPs were embedded, no evident changes in FTIR spectrum of composite fibrous membranes were observed. For example, the characteristic peak of Fe-O bond also appears at 557 cm^−1^ without obvious shift in the spectrum. However, we observed a slight shift at 1073 cm^−1^ in the composite fiber membrane, which means an increase in the hydrogen bond between the PU and γ-Fe_2_O_3_ NPs [26].

**Table 1:** Major mid-IR vibrational modes and the corresponding wave numbers

Description of vibrations	Wave numbers (cm^−1^)
-O-H stretching vibration of H_2_O	3347
-N-H stretching vibration	3328
-C-H stretching vibration	2919
>C=H stretching vibration	1729
-C-H asymmetrical flexing vibration	1704
AmidII band	1529
-C-O stretching vibration	1073
-(CH_2_) rocking	771
-Fe-O stretching vibration	557

**Fig. 6:**
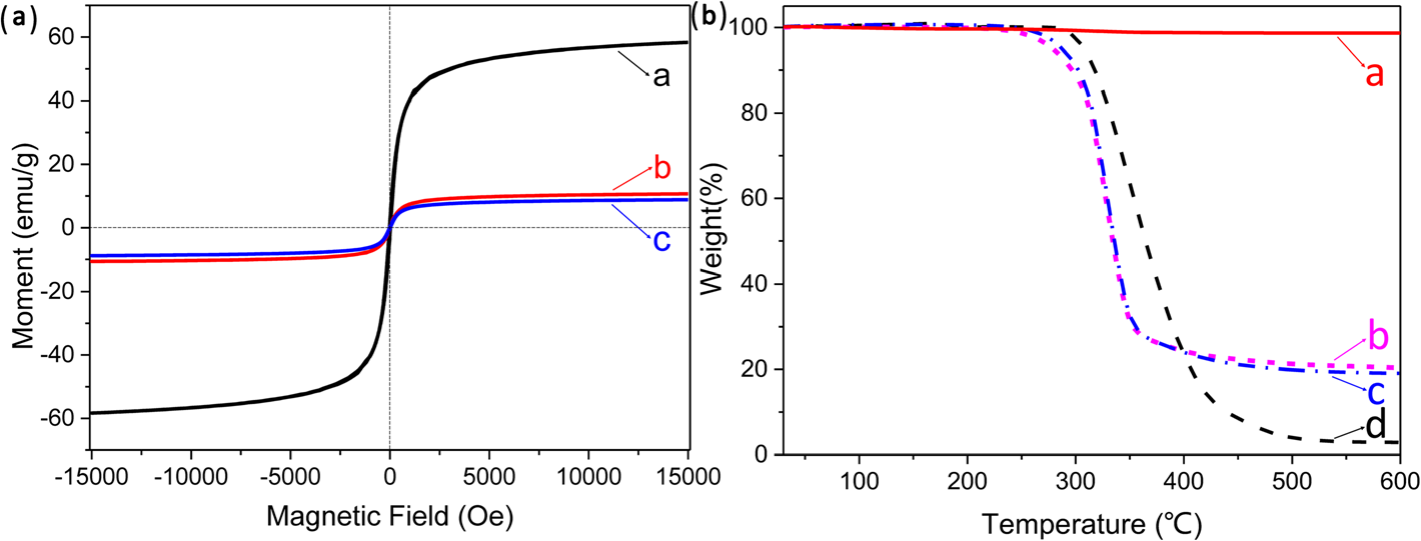
**a** Field-dependent magnetization curves of (*a*) γ-Fe_2_O_3_ NPs and γ-Fe_2_O_3_/PU composite fiber membranes prepared (*b*) with and (*c*) without the use of an auxiliary electrode at room temperature. **b** Thermogravimetric curves of (*a*) γ-Fe_2_O_3_ NPs, γ-Fe_2_O_3_/PU composite fiber membranes prepared (*b*) with and (*c*) without the use of an auxiliary electrode, and (*d*) pure PU electrospun fiber membranes

The magnetization curves of γ-Fe_2_O_3_ NPs and composite fibrous membranes prepared with/without an auxiliary electrode, as measured by VSM at 300 K, all revealed a typical superparamagnetic behavior with no distinct hysteresis loop and magnetization values of 58.3, 10.7, and 10.0 emu/g at 15,000 Oe, respectively, which shows that all the samples possess superparamagnetism (Fig. 6A) [5, 27]. The obvious decrease in the magnetization value of the two kinds of composite fibrous membranes in comparison with γ-Fe_2_O_3_ NPs at 15,000 Oe can be ascribed to the existence of nonmagnetic PU containing the magnetic nanoparticles and the unhomogeneous distribution of the magnetic nanoparticles in composite fibrous membranes [28, 29]. However, the magnetization values of the two kinds of composite fibrous membranes prepared using different electrodes show deviation from the theoretical value calculated by the doping ratio of γ-Fe_2_O_3_ NPs. The amount of γ-Fe_2_O_3_ NPs incorporated into the composite fibrous membranes can be estimated using the equation:

1 $$ \mathrm{Doping}\ \mathrm{ratio}\ \mathrm{of}\ \upgamma -{\mathrm{Fe}}_2{\mathrm{O}}_3\ \mathrm{nanoparticles}=\kern0.5em \mathrm{Mb}/\mathrm{Ma}\times 100\% $$

where Ma, Mb are magnetization value of pure γ-Fe_2_O_3_ nanoparticles and composite nanofibrous membranes at 15,000 Oe, respectively. According to Eq. (1), the actual doping ratios are about 18.3% and 17.1% in the composite membranes prepared with/without the auxiliary electrode. Besides the influences of both PU and the distribution of magnetic nanoparticles in composite fibrous membranes, the precipitation of γ-Fe_2_O_3_ NPs during electrospinning process also plays a critical role in the magnetization value. The precise measurement of the doping ratio of γ-Fe_2_O_3_ NPs can be further performed by the thermogravimetric analysis (TGA).

In order to confirm the weight ratio of γ-Fe_2_O_3_ NPs and thermal stability of composite fibrous membranes, the TGA was performed (Fig. 6B). As the curve a in Fig. 6B shows, the mass of γ-Fe_2_O_3_ NPs does not significantly decrease with increasing temperature. The initial thermal decomposition temperature (~ 260 °C) of the composite fibrous membranes is lower than that of the pure PU fibrous membrane (~ 305 °C), which completely satisfies the demanded thermal stability of the composite fibrous membranes for the magnetic heat treatment (curves b, c, and d in Fig. 6B). Then in the temperature range from 305 to 425 °C, the PU fibrous membranes show a steady degradation (curved in Fig. 6B). When the temperature reaches up to 500 °C, there is no obviously residual weight for the PU fibrous membrane in comparison to the composite fibrous membranes. It can be inferred from the residual fraction of composite fibrous membranes that the γ-Fe_2_O_3_ NPs doping in the composite membranes are 19.1 wt% and 20.4 wt%, which corresponds to the estimated results of VSM. Comparing the residual weight ratios of the composite fibrous membranes prepared with/without an auxiliary electrode, it is obvious that the addition of the auxiliary electrode does not affect the doping amount of the magnetic particles in the composite fibrous membranes. This small deviation of the embedded γ-Fe_2_O_3_ NPs can be attributed to the electrospinning process.

### In Vitro Hyperthermia Measurements

**Fig. 7:**
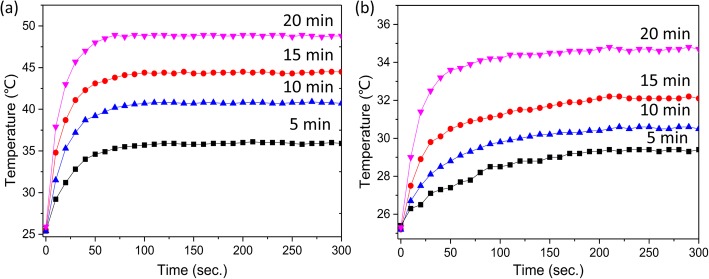
Temperature (T)-time (t) profiles for the electrospun fiber membranes prepared by in situ electrospinning **a** with and **b** without the use of an auxiliary electrode for 5, 10, 15, and 20 min upon the application of AMF

Magnetic nanoparticles hyperthermia utilizes the ability of the superparamagnetic γ-Fe_2_O_3_ NPs to generate heat under the action of high-frequency AMF [30]. The loss mechanism of the γ-Fe_2_O_3_ NPs under the AMF should be considered, respectively, whether the γ-Fe_2_O_3_ NPs are dispersed or aggregated. Although the heat generation of the aggregated γ-Fe_2_O_3_ NPs is determined by the hysteresis loss and the intermolecular interaction [31], the dispersed γ-Fe_2_O_3_ NPs are given by the relaxation of Brown and Néel [32]. And the γ-Fe_2_O_3_ NPs are incorporated and fixed inside the fibers, so the free rotation of γ-Fe_2_O_3_ NPs can be excluded, and Brown relaxation does not contribute to the magnetic heating that takes place under the AMF. For the embedded magnetic nanoparticles, only the hysteresis losses and Neel relaxation make a critical impact on the magnetic reversal loss heating. The actual AMF-dependent heat generation property of the γ-Fe_2_O_3_ NPs doped in polymer fibers is not easy to estimate due to the complex magnetic interaction of the mixed phases and the structure of composite fibrous membranes, the local aggregation, and the partial dispersion of the γ-Fe_2_O_3_ NPs [33]. Thus, the actual AMF-dependent heat generation property of the mixed structure of the dispersed and aggregated γ-Fe_2_O_3_ NPs in the fiber mat can be properly evaluated by experimental thermal behavior. Therefore, the magnetic conversion effect was performed by exposing composite fibrous membranes to an AMF. Figure 7 exhibits the time-dependent heating curves of pure PU fibrous membranes and different magnetic composite fibers. As shown in Fig. 7a, the temperature increase was 10.5 ± 0.4, 16.2 ± 0.3, 19.1 ± 0.5, and 24.4 ± 0.3 °C for γ-Fe_2_O_3_/PU-A5, γ-Fe_2_O_3_/PU-A10, γ-Fe_2_O_3_/PU-A15, and γ-Fe_2_O_3_/PU-A20 composite fibrous membranes, respectively. And in Fig. 7b, corresponding to the composite membranes prepared without the addition of an auxiliary electrode, the temperature increase was 4.2 ± 0.3, 5.1 ± 0.2, 6.7 ± 0.4, and 9.3 ± 0.2 °C for γ-Fe_2_O_3_/PU-5, γ-Fe_2_O_3_/PU-10, γ-Fe_2_O_3_/PU-15, and γ-Fe_2_O_3_/PU-20 composite membranes, respectively. It was observed that the heating temperature of all magnetic composite fibers increased rapidly with the increase of time and it appeared to eventually arrive at and basically maintain a balance at the end of the examination period. Also, a progressive increase in the heating rate and heating temperature emerged in both the composite fibrous membranes prepared by two different ways, as the time increased for preparing the magnetic composite fibrous membranes, and wherein the existence of γ-Fe_2_O_3_ NPs was confirmed by XRD diffraction and morphological analysis. However, at the same preparation time, the heating speed of the composite fibrous membranes prepared with the aid of the auxiliary electrode was faster and the stable temperature was higher than that of the other. For example, the heating rate of the composite fibrous membranes obtained by electrospinning for 15 min with the aid of the auxiliary electrode is 0.42 °C/s, and the equilibrium temperature is 44.3 °C. Moreover, if a fiber membrane having the same heating capacity is desired, a spinning time of 20 min is required with the aid of an auxiliary electrode, which means that the addition of the auxiliary electrode can remarkably improve the spinning efficiency and make full use of the spinning precursor. The results thus clearly indicate that the heating rate and the upper limit of the temperature rise are both remarkably improved compared to the composite membranes obtained without the aid of the auxiliary electrode. In contrast, the pure PU nanofibrous membranes showed slight temperature change under the identical conditions, which could be assigned to the influence of measurement error and the ambient temperature.

**Fig. 8:**
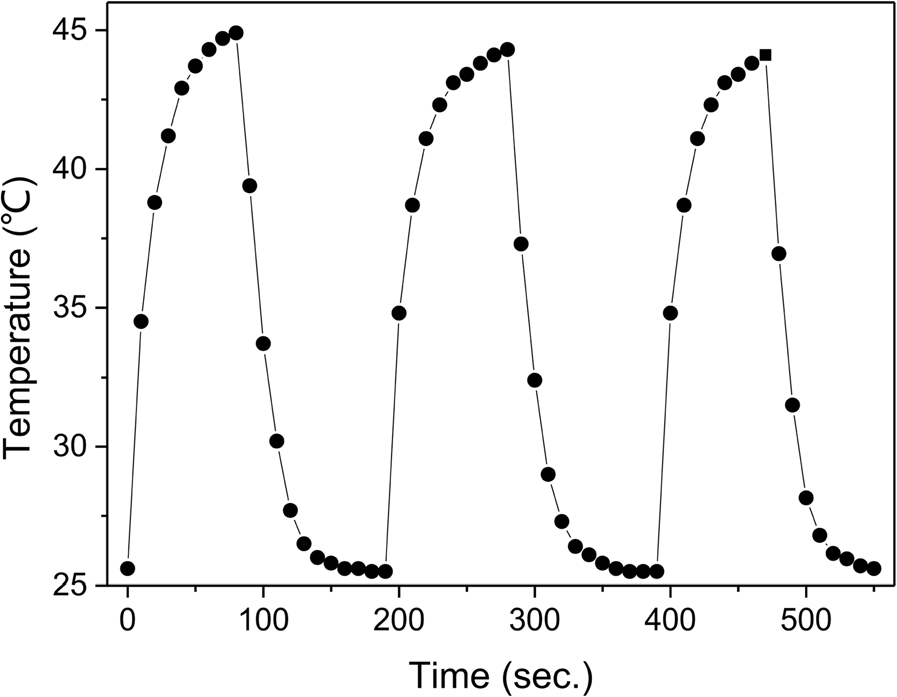
Cyclic heating profile of the electrospun fiber membranes prepared by in situ electrospinning

In the case of cancer therapy, the high- and low-temperature cycle of hyperthermia treatment is preferred along with other hyperthermia modes due to the chance of tumor metastasis, which means it is necessary for composite fibrous membranes to possess a uniform cyclic profile with a constant temperature rise during the heating process [34]. To test the heat stability of γ-Fe_2_O_3_/PU composite fibrous membranes, γ-Fe_2_O_3_/PU-A15 membranes were exposed to AMF for different cycles. As shown in Fig. 8, no obvious change in the elevated temperature profiles was observed during the three cycles of AMF effect, which indicated that the γ-Fe_2_O_3_/PU composite fibrous membranes could efficiently and rapidly convert AMF energy into thermal energy. More importantly, significant superiority of the composite fibrous membranes for cancer hyperthermia treatment was their capability for repeatable heating without damaging the heating efficiency.

**Fig. 9:**
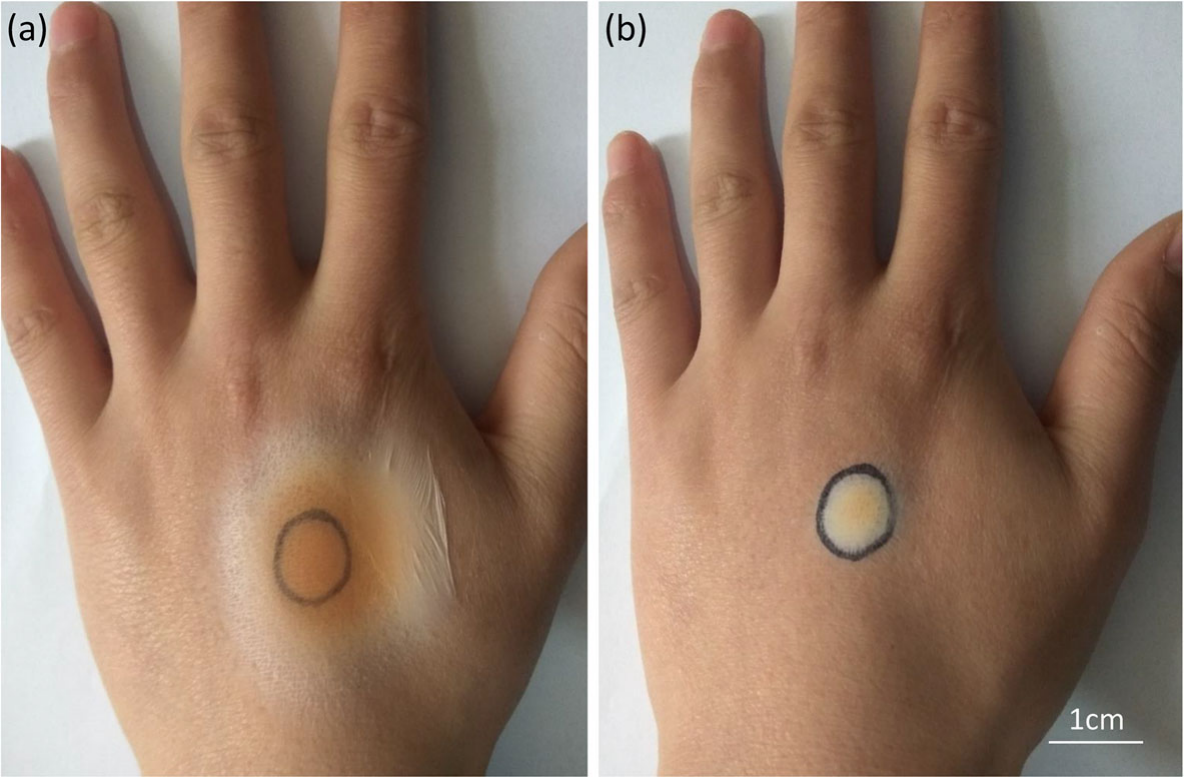
Schematic of in situ electrospun magnetic fibrous membrane on the surface of hand **a** without an auxiliary electrode and **b** with an auxiliary electrode

As mentioned above, the portable electrospinning device with the aid of an auxiliary electrode can quickly and precisely deposit the electrospun fiber membrane on the collecting pole in situ, which is in favor of the close contact between the prepared electrospun fiber membrane and the affected area, and improves the heating efficiency of the magnetocaloric therapy. Moreover, the thermotherapy fibers containing chemotherapeutic drugs can also be prepared in situ on the tumor tissue, which is beneficial to the synergistic effect of the drug and hyperthermia. As shown in Fig. 9, the electrospun fiber membrane can be prepared in situ on the surface of a hand. As can be found in Fig. 9a, a thin PU composite fibrous membrane is formed on the surface of the hand by a portable electrostatic spinner without an auxiliary electrode. Figure 9b shows that a tightly bonded, precisely deposited magnetic fibrous membrane is fabricated on the scar of the hand, which just like a second layer of skin due to the electrostatic attraction force. The mark has been completely covered by the magnetic fibrous membrane, while other skin tissue is not affected, which means a good versatility of the in situ preparation of magnetic fiber membranes under the assist of an auxiliary electrode.

## Conclusions

In summary, a magnetic composite nanofiber membrane was fabricated in situ using a portable electrospinning device with the aid of an auxiliary electrode. In the electrospinning process, the addition of the auxiliary electrode prolongs the stable area of the electrospinning and reduces the fiber whipping, thereby reducing the deposition range of the fiber and accelerating the fiber deposition rate. For electrospinning techniques, the application of conical auxiliary electrodes to precisely control the deposition area is suitable for most electrospinning materials. Moreover, the microstructure (diameter, surface morphology) of the electrospinning fiber is not significantly affected. The in situ prepared magnetic composite nanofibrous membranes can convert the AMF energy to the thermal energy to elevate temperature efficiently. With the aid of the auxiliary electrode, the composite fibrous membrane prepared by in situ electrospinning showed efficient heating ability upon the application of AMF, and well-maintained cyclic heating performance under the presence of AMF. These results indicate that the magnetic composite fibrous membrane prepared in situ by the auxiliary electrode is an excellent candidate for the magnetic hyperthermia of cancer therapy.

## Abbreviation

AMF: Alternating magnetic field
DMF: *N*,*N*-Dimethylformamide
FTIR: Fourier transformer infrared spectrometer
INOPs: Iron oxide nanoparticles
MES: Magnetic electrospinning
PU: Polyurethane
SEM: Scanning electron microscopy
TEM: Transmission electron microscope
TGA: Thermogravimetric analysis
VSM: Vibration sample magnetometer
XRD: X-ray powder diffraction
